# Circadian- and Light-Driven Rhythmicity of Interconnected Gene Networks in Olive Tree

**DOI:** 10.3390/ijms26010361

**Published:** 2025-01-03

**Authors:** Ivano Forgione, Tiziana Maria Sirangelo, Gianluca Godino, Elisa Vendramin, Amelia Salimonti, Francesco Sunseri, Fabrizio Carbone

**Affiliations:** 1 Research Centre for Olive, Fruit and Citrus Crops, Council for Agricultural Research and Economics (CREA), Via Settimio Severo 83, 87036 Rende, CS, Italy; 2 Research Centre for Olive, Fruit and Citrus Crops, Council for Agricultural Research and Economics (CREA), Via di Fioranello 52, 00134 Roma, Italy; 3 Department Agraria, University Mediterranea of Reggio Calabria, Località Feo di Vito, 89124 Reggio Calabria, Italy

**Keywords:** *Olea europaea* L., circadian clock (CC), continuous light (LL), targeted RNA sequencing, photoreceptors, fruit pigments, FAs, development, breeding

## Abstract

A circadian clock (CC) has evolved in plants that synchronizes their growth and development with daily and seasonal cycles. A properly functioning circadian clock contributes to increasing plant growth, reproduction, and competitiveness. In plants, continuous light treatment has been a successful approach for obtaining novel knowledge about the circadian clock. The olive tree (*Olea europaea* L.) is one of the most important crops in the Mediterranean area, and, so far, limited information is available on its CC gene network. Here, we studied the behavior of circadian rhythm genes under LD (light/darkness) and LL (light/light) conditions, the relationships in this network, and the ability of the treatments to modulate gene expression in the photoprotective pigment and lipid biosynthesis pathways. One month of LL conditions increased olive growth performance, but LL exposure also caused reductions in vegetative growth and chlorophyll accumulation. A panel was designed for a study of the transcription expression levels of the genes involved in light perception, the CC, and secondary metabolite and fatty acid biosynthesis. Our results revealed that the levels of 78% of the transcripts exhibited intraday differences under LD conditions, and most of them retained this rhythmicity after exposure to one and two months of LL conditions. Furthermore, co-regulation within a complex network among genes of photoreceptors, anthocyanidins, and fatty acids biosynthesis was orchestrated by the transcription factor HY5. This research enriches our knowledge on olive trees grown under prolonged irradiation, which may be attractive for the scientific community involved in breeding programs for the improvement of this species.

## 1. Introduction

Light is an essential environmental factor in plant life. It is converted into chemical energy via photosynthesis [1], which is the engine for plant growth and development. The duration, intensity, and wavelength of the light are the bases of many physiological processes. Studies on prolonged photoperiods of up to 24 h irradiation have found contrasting effects on different plant species. Positive effects, such as the increased production of fresh and dry matter in *Eruca vesicaria* (L.) Cav. [2], greater leaf size and accumulation of chlorophyll in *Vigna radiata* L. [3], acceleration of the reproductive cycle in long-day plants [4], and increases in the anthocyanins and flavonoids contents in *Brassicaceae microgreens* [5], were observed. In contrast, negative responses to 24 h light exposure in tomato and eggplant, such as visible leaf chlorosis after few days of treatment with the hypothesis of being due to starch overaccumulation preventing photosynthesis [6,7], were also observed.

Plants, bacteria, and animals possess special proteins called photoreceptors that are covalently bonded to a chromophore that has a light-absorbing function [8,9,10]. Photoreceptors are able to sense light and mediate the triggering of signal transduction pathways that finally cause a biological response [11]. The phytochromes (PHYs) are red and far-red light receptors [12], whereas the cryptochromes (CRYs) respond to UV-A and blue light [13] as well as phototropins (PHOTs) [14]. The mRNA and protein abundances of phytochromes and cryptochromes are responsible for modulating the plant circadian oscillator [15]; in turn, the circadian regulates the mRNA and protein levels of both types of photoreceptors in a feedback loop [16]. The circadian clock is a natural 24 h rhythm system evolved for adaptation to daily and seasonal variations, which works as a linker between internal programs and external stimuli from the environment such as light [17]. In *Arabidopsis*, the main components of the circadian oscillator belong to the family of the MYB transcription factors (TFs) LHY (late elongated hypocotyl) and CCA1 (circadian clock associated 1), the induction of which is quickly light-mediated at dawn. At dusk, LHY and CCA1 protein levels decrease [18,19], and the family of TOC1 (timing of cab expression 1), which comprises pseudo-response regulators, is expressed for a few hours [20]. LHY and CCA1 are thought to repress *TOC1* expression by binding evening element (EE) motifs within their promoter regions. In turn, TOC1 inhibits *LHY* and *CCA1* transcription by engaging with their promoters [21]. The zinc finger protein Constans (CO), which is modulated by these circadian clock genes [22,23], is required to induce flowering under long-day conditions as an activator of the floral integrator *flowering locus T* (*FT*) [24]. Continuous light is known to alter the expression of the circadian clock gene components [25].

Although many studies have been conducted on these mechanisms in herbaceous plants and partly on woody species, limited information is available for olive trees. In particular, studies of angiosperms and gymnosperms, such as *Populus* spp. and *Picea abies*, respectively, have elucidated many aspects of the daily and seasonal control of plant growth. In detail, a major role of photoperiod perception has been found in the pause in plant growth during the autumn as well as in the resumption of shoot growth during the spring [26,27,28,29,30].

The role of light in controlling the production of photoprotective pigment, and lipid biosynthesis has been recently studied in the olive tree by combining metabolomic and transcriptomic strategies [31]. The RNA-seq approach has often been employed to investigate the expression of key pathways in response to light to obtain an overview on transcript behavior, especially of the circadian clock genes [32,33,34]. In detail, time-course circadian transcriptome profiling offers a comprehensive approach for identifying a wide array of circadian rhythm genes [35,36,37,38,39]. The advances in the genomic knowledge of olive trees and the availability of reference genome assemblies for both wild and cultivated varieties [40,41,42,43,44,45,46] have promoted studies aiming to isolate and functionally characterize genes using integrated approaches (transcriptomics, proteomics, and functional studies).

So far, in olive trees, the relationship between light and circadian clock machinery has been entirely unexplored. Herein, the RNA sequencing of a panel of specific known genes was employed to gain knowledge on the expression of the circadian clock genes in the long-day species *Olea europaea* L. and how they coordinate, in combination with photoreceptors, the expression of fatty acids and polyphenol genes by their expression under LD (light/darkness) and LL (light/light) conditions.

This study sheds light on the olive tree’s ability to maintain the intraday differences in circadian clock genes and their related interactors under an LL regime. The maintenance of this expression of circadian rhythm genes for several months could provide a valuable resource for future investigations.

## 2. Results

### 2.1. Plant Growth Measurements

**Figure 1:**
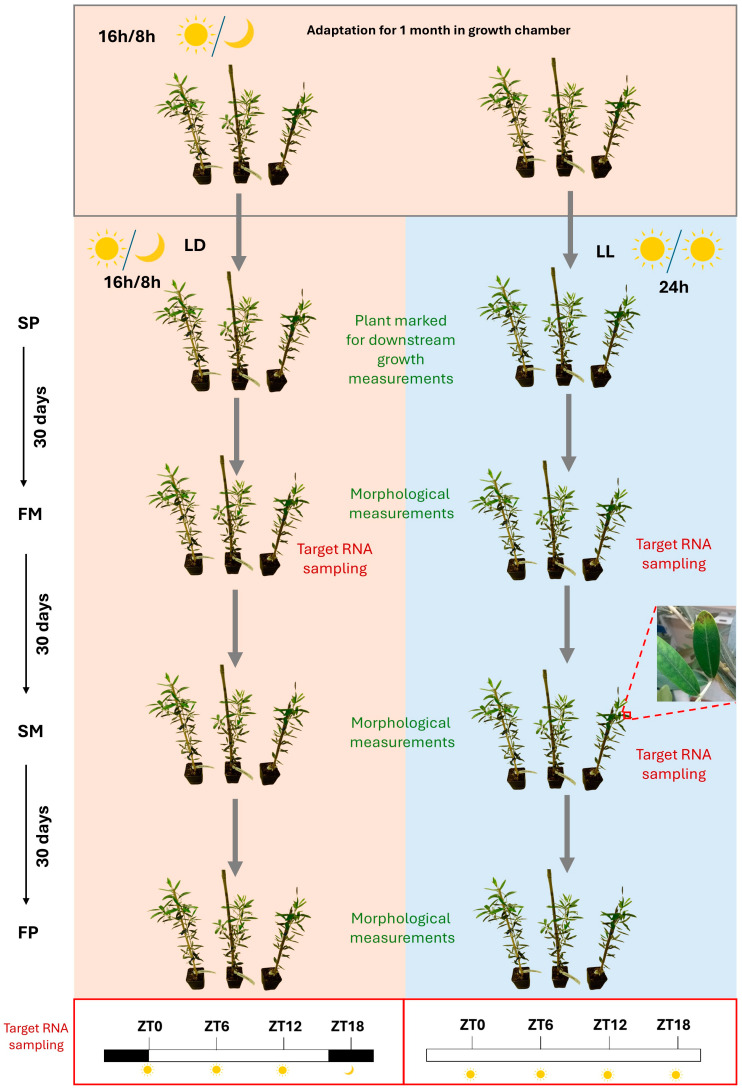
Graphical representation of the experimental design, where stressed leaves are shown after 2 months of LL (light/light) treatment. Start point (SP), 30 days from SP—first midpoint (FM), 60 days from SP—second midpoint (SM), and 90 days from SP—final point (FP). Sampling for the RNA-seq was performed every 6 h for 24 h considering the Zeitgeber time (ZT0, ZT6, ZT12, and ZT18), where ZT0 represents the presumed dawn.

To increase the knowledge about the growth of young ‘Leccino’ olive plants exposed to different photoperiods, several parameters were measured after exposure to LD (light/darkness) and LL (continuous light) regimes (Figure 1 and Figure S1).

**Figure 2:**
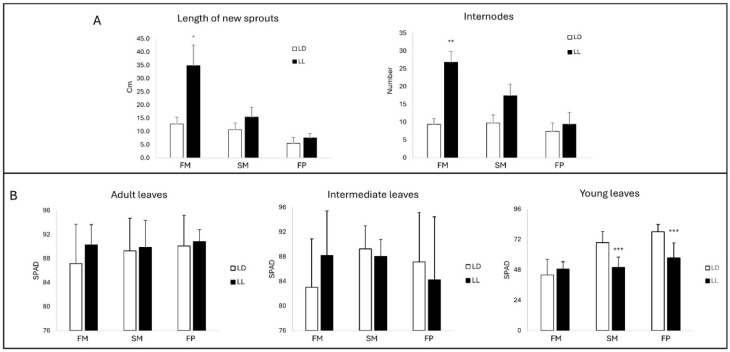
Vegetative growth in terms of length of new sprouts (cm) and number of internodes (**A**) and chlorophyll content in adult, intermediate, and young leaves (**B**) were measured at one (FM), two (SM), and three (FP) months from the experimental start (SP) in plants grown under LD (light/darkness) and LL (light/light) conditions. The results are presented as the mean value ± standard error. Asterisks indicate significant pairwise differences according to Student’s *t*-test (* *p* ≤ 0.05, ** *p* ≤ 0.01, *** *p* ≤ 0.001).

The length of the new sprouts and the development of new nodes was higher by about three-fold after 30 days (FM) under LL compared to LD conditions (Figure 2A, Table S1). Further exposure for 30 (SM) and 60 days (FP) to the LL regime conferred a higher rate vegetative growth in terms of cellular proliferation and elongation in the plants, but the length of new sprouts and the new nodes formed did not show significant differences compared to the LD regime (Figure 2A, Table S1). The chlorophyll content was measured to obtain information on the photosynthetic capacity of the olive plant under different photoperiod regimes. In both conditions, adult leaves accumulated higher amounts of photosynthetic pigments than intermediate and young leaves (Figure 2B, Table S2). Although a significant difference was not observed in the LD vs. LL comparison, the mean value of chlorophyll was higher in mature leaves in the LL regime at the FM, SM, and FP. In intermediate leaves, limited differences in chlorophyll content were detected between the LD and LL regimes at any time point, whereas, in young leaves, a significant increase was observed in LD vs. LL conditions at the SM and FP (Figure 2B, Table S2). A reduction in the photosynthetic process was observed only after the extension of LL conditions to the second month in the neo-formed, not the older, tissues, suggesting that anatomical differences in the leaf layers may occur in plants grown under LL conditions, and these may affect the competence of these organs at light perception level. Moreover, after exposure for two months (SM) to LL conditions, the plants looked sick with yellow leaves; these symptoms were a sign that they were being stressed by the prolonged exposure to the LL regime (Figure 1 and Figure S1).

### 2.2. Transcriptional Profiling Using the Olive-Targeted RNA Panel

Morphological trait analysis suggested that the best performance in terms of vegetative growth was achieved after one month under LL conditions; early symptoms of stress appeared after two months under the same regime. Thus, we focused on plants exposed to 30 (LL-FM) and 60 (LL-SM) days of LL compared to LD conditions for gene expression analysis. By using a panel aimed to target genomic regions of interest [31], the RNA-seq of the specific genes involved in the phenylpropanoid pathway, fatty acid metabolism, photoperception, and the circadian clock machinery was carried out. The high-quality filtered sequenced reads were aligned to the ‘Leccino’ reference genome (v. 4, http://olgenome.crea.gov.it/index.php?lang=en, accessed on 1 January 2025). The assembly of ‘Leccino’ had not yet been published; therefore, the targeted sequences and described genes were reannotated. As a consequence, in all the tables and figures, we report the fully updated functional annotations for each gene according to BLAST analysis; the full-length sequences of the targeted genes are provided (Table S3, Sequence S1). The alignment percentages of the mapped reads on the reference genome were comparable within replicates and cultivars (Table S4).

#### 2.2.1. Diurnal mRNA Oscillations of Interconnected Networks

**Figure 3:**
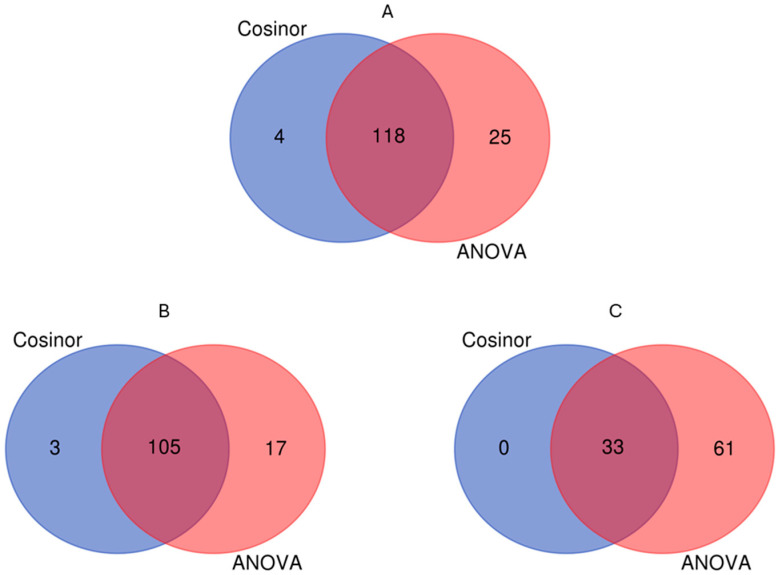
Venn diagram of significantly different loci detected by Cosinor and ANOVA under LD (**A**), LL-FM (**B**), and LL-SM (**C**) conditions.

To describe the patterns of cyclical activities such as diurnal cycles and circadian rhythms, we scored the expression values of the 187 targeted genes using both Cosinor and one-way ANOVA in our analyses [47]. The sequencing data obtained from the olive plants grown under LD conditions showed that 147 genes out of 187 had a *p*-value ≤ 0.05 for at least either Cosinor analysis or one-way ANOVA and were thus defined as “diurnal genes” (Table S5, Figure 3A).

**Figure 4:**
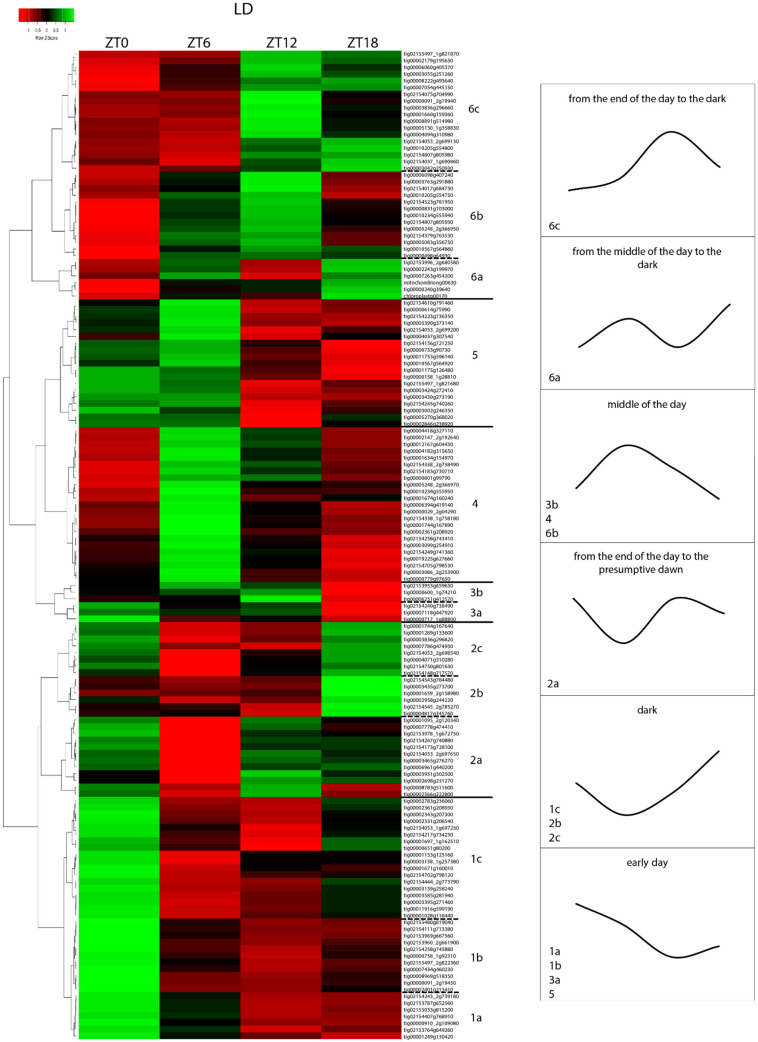
The heatmap includes 147 loci that exhibit significant oscillation intraday under LD conditions according to Cosinor and/or ANOVA. The color intensity represents the mean value of three replicates of the gene expression at Zeitgeber time (ZT) 0, ZT6, ZT12, and ZT18. The dendrogram indicates the relatedness of genes and helps to identify different clusters. The curves represent the six main different expression patterns formed by the genes belonging to the clusters indicated within each frame.

To identify genes that were similarly expressed, we implemented and evaluated an average-linkage clustering analysis through Pearson correlations of the 147 “diurnal genes”. According to this analysis and based on the expression of a single locus, six different macro-clusters were identified. The colored blocks in the heatmap distinguish the clusters (Figure 4), and the dendrogram branches were considered groups of loci with common expression.

In the first macro-cluster (1), we identified three different patterns that shared high expression at presumptive dawn (ZT0) an negative regulation at the end of the day (ZT12). In the first pattern, named 1a, the peak at ZT0 was followed by a persistent upregulation early in the day; 1b showed upregulation exclusively at dawn; and, in 1c, the gradual increasing expression of the genes was visible during the dark, with a clear peak at ZT0. The three patterns identified for the second macro-cluster (2) shared a trough early in the day: 2a showed upregulation at ZT0, which was followed a signal for upregulation at the end of the day and during the dark; in 2b, they were upregulated in the dark; in 2c at ZT0, a tail of the peak detected at ZT18 was visible. Six loci with different patterns of expression were included in the third cluster (3): 3a showed upregulation in the early hours of the day, while 3b were induced in the central hours of the lighting period. The fourth macro-cluster (4) comprised genes with lowest expression in the night and highest at ZT6. The fifth macro-cluster (5) was very similar to 1a with a weak activation of the transcription observed at ZT0 followed by a peak at ZT6. In the sixth macro-cluster (6) were included the genes sharing a down-regulation at dawn and an induction from ZT6 onwards: 6a showed two clear peaks at ZT6 and ZT18, 6b exhibited upregulation in the central hours of the day (such as macro-cluster 4), while 6c peaked at ZT12 with persisting upregulation during the dark and low expression early in the day.

In the scenario of alternating light and dark exposure, the majority of the genes showed a similar pattern of expression, giving rise to genetic networks that clearly turn on at specific hours of the day. Here, we grouped the genes that shared similar expression patterns by focusing on their up- and downregulation during 24 h.

##### Early and Daytime Gene Expression

Firstly, the 40 genes highly transcribed in early morning with an upregulation from dawn onward included in clusters 1a, 1b, 3a, 5 were considered. Among them, genes were identified that encode the photosystem I reaction center subunit III (PSAF), photosystem I reaction center subunit (PSAK), and photosystem I reaction center subunit N (PSAN) subunits in the reaction center of photosystem I; photosystem II 22 kDa protein (PSBS) and photosystem II reaction center W protein (PSBW) subunits of the photosystem II; and for light-harvesting chlorophyll A-B binding protein (LHCB3 and LHCB5).

At this time, the genes encoding the red/far-red photoreceptor phytochromes (PHYB, PHYE) and blue-light photoreceptors such as cryptochrome 1 (CRY1) and proteins containing an LOV domain, which confers blue-light photoreceptor activity, such as the PAS/LOV protein (PLP), were highly expressed.

In the early morning, several transcription factor (TF) genes were also upregulated: *Phytochrome Interacting Factor* 3 (*PIF3*) and *PIF4*, related to the red/far-red light photoreceptors; a key component of the circadian clock, *late elongated hypocotyl* (*LHY*) of the MYB family; two *Reveille 6* (*RVE6*) genes; *RVE8* involved in the circadian clock; four members of the zinc finger *Constans-like* family (*COL*), *COL1*, *COL2*, and *COL4*; *B-BOX protein 14* (*BBX14*), which have a key role in flowering; as well as subunits A (*SIGA*) and E (*SIGE*) of chloroplast RNA polymerase.

With similar patterns of expression, seven genes encoding enzymes for fatty acid biosynthesis, including 3-ketoacyl-CoA synthase 6 (KCS6), KCS11, KCS19, biotin carboxyl carrier of acetyl-carboxylase (BCCP), glycerol-3-phosphate acyltransferase (GPAT1), GPAT9, and acyl-ACP thioesterase (ACPTE), were identified.

At ZT0 and ZT6, the overexpression of seven significantly different genes related to the biosynthesis of anthocyanidins suggested that this time of the day regulates this specific cascade of reaction: four *Phenylalanine Ammonia-Lyase* (*PAL*), *4-Coumarate-CoA Ligase 1* (*4CL*), *Dihydroflavonol 4-Reductase* (*DFR*), *Putative Anthocyanidin Reductase* (*ANR*), *Flavone Synthase II* (*FNS*) of flavones, *Flavonol Synthase* (*FLS*) of flavonols, and *Cinnamoyl Reductase* (*CCR*) of lignin.

Notably, the gene annotated as *Long Hypocotyl 5* (*HY5*) exhibited flat expression during light hours, with a low in the night under LD conditions and was thus included in the early day group, corresponding to the beginning of its induction.

##### Gene Expression During the Dark

Upregulation in the early morning also occurred in an additional group of 32 loci that were also highly transcribed at ZT0, but they were characterized by being activated signal in the dark before dawn, as observed in clusters 1c, 2b, and 2c; in contrast, the maximum expression of the genes included in cluster 2b was reached at ZT18, including genes encoding for Photosystem I Reaction Center Subunit IV (PSAE) and Photosystem I Assembly Factor PSA3 (PSA3) in the reaction center of the photosystem I; Photosystem II Repair Protein, CRY1, and phototropin 2 (PHOT2), which showed similar patterns; as well as PIF1, Constans, Constans-like, Timing of Cab Expression 1 Domain-Containing Protein 101 (CCT101), and Flowering Locus D (FLD).

Upregulation was found between ZT18 and ZT0 for 14 genes encoding for enzymes of the fatty acids biosynthesis pathway such as GPAT1, GPAT3, GPAT9, three BCCP, ACPTE, and an alpha subunit of the acetyl-coenzyme A carboxylase carboxyl transferase (ACC). Furthermore, KCS6, two Very-Long-Chain Enoyl-CoA Reductase (ECR), a gene encoding Diacylglycerol Acyltransferase 1 (DGAT1), a gene encoding Stearoyl-[Acyl-Carrier-Protein] 9-Desaturase (SACPD), as well as Enoyl-[Acyl-Carrier-Protein] Reductase (EAR) exhibited upregulation at ZT18 and ZT0.

A co-expression of the genes related to phenylpropanoids was observed, and nine genes exhibited an expression profile that partially overlapped that of the others that were upregulated early in the day. At ZT18 and ZT0, three *PAL*, *Chalcone Isomerase* (*CHI*), both mapped *Trans-Cinnamate 4-Monooxygenase* (*C4H*), *ANR*, *Naringenin, 2-Oxoglutarate 3-Dioxygenase* (*F3H*), and *Cinnamoyl-Reductase 1-like* (*CCR1*) in lignin biosynthesis were significantly upregulated.

##### Gene Expression from the End of the Day to the Presumptive Dawn

Twelve genes belonging to cluster 6c showed a particular expression pattern with the activation of transcription at ZT12 that persisted throughout the night, reaching a maximum level of expression with turning on of the light. Here, a severe low at ZT6 was observed. This expression pattern occurred for *LHCA2.1*, the unique mapped *CRY2*, *XAP5 Circadian Timekeeper* (*XCT*) and *Early Flowering 3* (*ELF3*).

The five genes encoding the fatty acids that exhibited the same pattern of expression were Biotin Carboxylase 1 (BC1), ECR, ACC subunit alpha, DGAT2, and *3*-oxoacyl-[acyl-carrier-protein] synthase *3.*

Genes such as *Leucoanthocyanidin Dioxygenase* (*LDOX*), *DFR*, and *FLS4*, involved in the phenylpropanoid pathway, followed this expression pattern.

##### Gene Expression from the End of the Day to the Dark

The turning off of the light represent4r a key point for the activation of the biological function of some light-dependent genes. Eighteen genes exhibited an induction at dusk between ZT12 and ZT18. Among them, there were a gene encoding the PHYA and UVB-Resistance 8 (UVR8) of the photoreceptors; two components of the PHYA signaling network, Far-Red Elongated Hypocotyl3 (FHY3) and Far1-Related Sequence 5-like (FRS5); two out of three genes of the evening-phase component of the circadian clock, *TOC1/PRR1* and *Pseudo-Response Regulator 7* (*PRR7*); two B-Box zinc finger transcription factors, *BBX13* and *COL6*; and *SIGF*.

Among the eighteen genes, five were related to fatty acid biosynthesis enzymes such as two ACC subunit alpha of the malonyl-CoA biosynthesis pathway, KCS4, 3-Oxoacyl-[Acyl-Carrier-Protein] Synthase 3, and DGAT1.

Similarly, two genes were involved in phenylpropanoid biosynthesis such as *Anthocyanidin 3-O-Glucosyltransferase 2* (*3GGT*) and *Flavonol Synthase/Flavanone 3-Hydroxylase* (*F3H*).

##### Gene Expression in the Middle of the Day

The genes included in clusters 3b, 4, and 6 were induced in the middle of the light period, between ZT6 and ZT12, such as *PSBR*; most of the mapped *LHCs: LHCA1*, *LHCA2*, and *LHCA3*, which are related to photosystem I; and *LHCB2*, *LHCB5*, *LHCB6*, and *LHCB7,* associated with photosystem II.

At the same time, one of the two mapped *PHYC* genes and one *PLP* among the photoreceptors were induced. Furthermore, *PIF3*, one of the two mapped *Far-Red Impaired Response 1* (*FAR1*), two *Suppressor of PhyA-105* (*SPA2*), and a *SPA4*, together with the two detected circadian-associated transcripts, annotated as *PRR5* and *ELF4*, were overexpressed for the regulation of the circadian clock.

In the middle of the day (between ZT6 and ZT12), genes involved in fatty acid biosynthesis such as *MBOAT Bound O-Acyl Family Protein*, *Acyl-CoA Binding Protein 4* (*ACBP4*), *ECR*, *SACPD*, and *KCS7* as well as two *KCS11* genes were upregulated. A gene related to phenolic compound biosynthesis, *Caffeoyl-CoA O-Methyltransferase* (*CCoAOMT*), implicated in the biosynthesis of lignin, was also highly transcribed.

Another thirteen genes from cluster 6b showed a different expression pattern from to the others already described. Their transcription was activated at ZT6, with a peak at ZT12 and a weak signal at night. This pattern of expression was observed for three photoreceptors containing an LOV domain: *PHOT1*, *Adagio3* (*ADO3*), and *Zeitlupe* (*ZTL*), as well as in *SIGC*, the other *TOC1/PRR1,* the flowering regulators in long-day *Gigantea* (*GI*) as well as the b-box zinc finger transcription factor *COL10*. The remaining four genes with similar expression behavior are related to enzymes of the fatty acid biosynthesis pathway, such as two KCS11 enzymes, Delta(12)-Fatty-Acid Desaturase (FAD2), Lysophospholipid Acyltransferase 1 (LPAT), SACPD, and BCCP.

Finally, the few genes included in cluster 6a displayed a sinusoidal expression pattern by exhibiting two peaks within 24 h at ZT6 and ZT18: two *Photosystem II Protein D2* (*PSBD*) genes, *CRY3*, *KCS11*, *Chalcone Synthase* (*CHS*), and *Leucoanthocyanidin Reductase* (*LAR*).

#### 2.2.2. Circadian Rhythmicity of Olive Transcripts Under LL Regime

##### Circadian Rhythmicity After One Month Under LL Regime

The sequencing data from the samples from the plants grown under LL conditions for one month revealed that 125 loci out of the 147 diurnal rhythmic loci showed significant rhythmicity according to the Cosinor algorithm and ANOVA, with a *p*-value < 0.05 (Table S6; Figure 3B).

**Figure 5:**
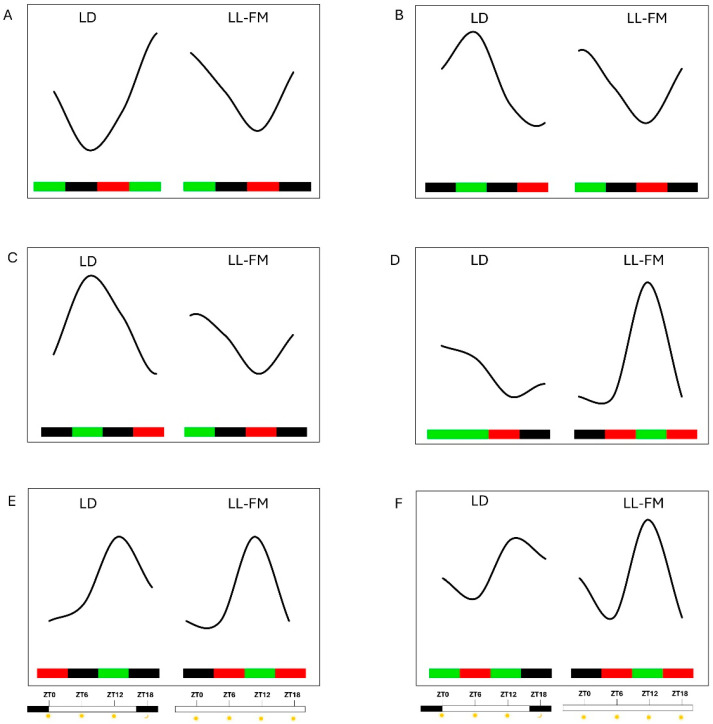
Gene expression pattern comparison between LD (light/darkness) and LL-FM (light/light) conditions at various Zeitgeber times (ZT0, ZT6, ZT12 and ZT18), where ZT0 represents the presumptive dawn. (**A**) Peak Expression (PE) at ZT0 and ZT18 in both LD and LL-FM; (**B**) PE at ZT0 and ZT6 in LD and at ZT0 and ZT18 in LL-FM; (**C**) PE at ZT6 in LD and at ZT0 and ZT18 in LL-FM; (**D**) PE at ZT0 and ZT6 in LD and at ZT12 in LL-FM; (**E**) PE at ZT12 in both LD and LL-FM; (**F**) PE at ZT12 and ZT18 in LD and at ZT12 in LL-FM. Green, black, and red blocks represent high, medium, and low expression, respectively.

After one month of LL exposure, a subset of genes was found that maintained the LD expression trend over the four time points. Among them, there were many genes related to photosystems that, under the LD regime, were induced from the night to the early morning, such as *PSA3*, *PSAF*, *PSAN*, *PSBS*, and *Photosystem II Repair Protein*. Under LL-FIP, we confirmed their maximum expression level at ZT0 and an evident downregulation near the presumptive dusk (photosystem-like pattern). Similarly, the genes for photoreceptors such as *PHYB*, both *PHYE* genes, both *CRY1* genes, and *PHOT2* followed the photosystem-like expression pattern, maintaining the same trend under LL as under LD conditions; as did *SIGE*; the partially redundant transcriptional activators within the circadian system, *RVE6* and *RVE8* the flowering regulators *COL4* and *FLD*; the fatty acid genes encoding for DGAT1, GPAT, GPAT9, and DFR in the anthocyanin pathway (Table S6; Figure 5A).

Interestingly, one month under LL conditions caused a different pattern of gene expression compared with the LD regime in the *LHCs*, such as *LHCA3*, *LHCB5*, *LHCB6*, and *LHCB7*, with their expression level increasing from ZT0 to ZT6 under LD but not under LL-FIP conditions. Under the LL regime, these genes were progressively downregulated from ZT0 to ZT12, with a re-induction at ZT18 (Table S6; Figure 5B). A similar pattern of gene expression was found for *PHYC*, *PLP*, *SIGA*, *PIF3*, *PIF4*, *SPA2*, *SPA4*, *FRS5*, *PRR5*, and *COL16* (Table S6; Figure 5B).

Another group of genes (*SIGC*, *PHOT1*, *ZTL*, *FHY3*, another *FRS5*, *GI*, two *TOC1*, a *PRR7*, *FAD2*, and *3GGT*) exhibited a photosystems-like pattern at the FM under LL conditions, being downregulated between ZT6 and ZT12 compared to their significant upregulation under the LD regime. Similarly, other genes showed a peak in expression in the night under LD conditions, such as the *PSBD*, *SIGF*, *PIF1*, and *TOC1*, which also exhibited the photosystems-like pattern under continuous light (Table S6; Figure 5C).

A large group of genes were significantly upregulated at ZT12 after one month under the LL regime. Some of them were upregulated in the early morning under LD conditions such as *PSAE-2* and the transcription factors *PIF3*, *COL2*, and *CCT101*. In addition, genes encoding for KCS19, two KCS6, KCS7, four KCS11, SACPD, ACC subunit alpha, EAR, BCCP, GPAT9, ACPTE, CCoAOMT, and ACBP4 exhibited this pattern. Furthermore, *HY5* exhibited flat expression during the light hours with a low in the night under LD conditions; under the LL-FIP regime, it was strongly induced at ZT12 (Table S6; Figure 5D).

A marked overexpression at ZT12 under the LL regime was also detected for genes related to phenylpropanoids in the early morning: two *PAL*, two *C4H*, *4CL*, *CHI*, *FLS*, and *FNS* (Table S6; Figure 5D).

At the FM under the LL regime, peaks in *KCS11*, *PHYA*, and *UVR8* were observed at ZT12; peaks were also observed only for *PHYA* and *UVR8* under LD conditions. Additionally, the genes encoding for fatty acids that were upregulated at the end of the lighting phase under LD, such as two *KCS11*, *GPAT1*, two *BCCP*, *F3H*, and *ECR,* showed the peak at ZT12 in LL-FM. In addition, a peak at ZT12 under LL-FM conditions was also observed for another mapped *ECR*, *BC1*, and *ACPTE*, which showed a steady level of expression under LD conditions (Table S6; Figure 5E).

Finally, phenylpropanoid-related genes were also strongly induced at ZT12 under the LL regime: *F3H* and a putative *DFR* maintained the same pattern under LD conditions, whereas two *PAL*, *CHS*, *LAR*, and *ANR* under LD conditions were expressed more at night (Table S6; Figure 5F).

##### Circadian Rhythmicity Reduced After Two Months Under LL Conditions

One additional month of exposure to the LL regime revealed that 94 of the 125 loci under LL-FM conditions exhibited significant intraday differences at the four time points analyzed (Table S7; Figure 3C). The pathways of red/far-red light perception and signaling were severely affected by prolonged continuous light because 8 out of 15 rhythmic loci related to this process remained significant under LL-SM conditions as well as 4 out of 9 loci of the circadian clock, and 3 out of 6 genes of *KCS11*. Moreover, the intraday expression pattern of a large number of loci under LL-SM conditions differed in comparison to under LL-FM conditions. Around 75% of the genes that peaked at ZT0 under LL-SM conditions showed the highest mRNA abundance at ZT12 under LL-FM conditions. All loci, except for a small number of genes, that exhibited significant downregulation at ZT0 under LL-SM conditions ha lowest expression at ZT12 under LL-FM conditions. The transcriptomic profile comparison of LL-FM vs LL-SM revealed a co-regulation of these two blocks of genes, although a shifted pattern of gene expression was observed.

## 3. Discussion

The plant circadian network has been broadly investigated for annual plants, with a huge number of papers published in the last two decades; on tree species, this topic has been studied mostly in poplar [29,48,49]. To the best of our knowledge, this is the first research carried out on the olive tree, which elucidated the behavior of the genetic circadian clock network and the related oscillating genes, which was also evaluated under the pressure caused by prolonged light irradiation. The experimental design, which is based on target RNA sequencing, represents a new approach to conventional total RNA-seq to investigate gene expression. In the latter, the interpretation of information to obtain clear results may be difficult given the large amount of data produced. Here, through targeted RNA sequencing, we focused on a restricted panel of 187 target genes previously retrieved from model species that showed a putative oscillating pattern, including 29 and 60 genes involved in the phenylpropanoid pathway and fatty acid metabolism, respectively. Moreover, the panel included 98 additional genes playing a role in photoperception and the circadian clock machinery, with key regulatory functions in the biosynthesis of secondary metabolites.

### 3.1. Photosynthetic Machinery Assembly and Anthocyanin Accumulation Mediated by Red- and Blue-Light Photoreceptors

Interestingly, around 65% of the target genes showed rhythmic expression under LD conditions; most of them conserved their intraday oscillation following 30 and 60 days of continuous light. The analysis of the expression pattern of these genes allowed us to identify intricate crosstalk among many of them in the olive tree. The experimental design with a time-course study highlighted that the red- and blue-light photoreceptors function in activating the transcriptional signaling of *HY5* to promote the photosynthetic machinery assembly. The light-dependent control of plant growth and development is finely regulated by many physiological and biological processes driven by the bZIP HY5 transcription factor, which binds thousands promoter genes and acts downstream of multiple photoreceptors [50,51,52]. Recent studies showed that the downregulation of this gene and the low accumulation of the protein HY5 occurs in *phyB1B2* and *cry1a* tomato mutants, opposite to what is observed in the *CRY1a-OX* and *phyA* lines [50]. These findings coincide very closely with ours, where the increased co-expression of *CRY1*, *PHYB*, and *HY5* was observed in the early morning, indicating that *HY5* is involved in the mechanism of regulation that is driven by red/far-red and blue photoreceptors. To further support the data obtained by Dong et al. (2023), who found that *HY5* was induced in a *phyA* mutant, we found that when *PHYA* was upregulated at the end of the day and at the night (ZT12–ZT18), *HY5* decreased. Moreover, in our previous study, we observed that *CRY1* upregulation was related to an increase in phenylpropanoid levels, which limited the repressor effect on the transcription factors *SPA1* and *HY5* [31].

Under LD conditions, all mapped members of the multigene families *LHCA* and *LHCB* showed a waveform expression with a peak between ZT6 and ZT12; therefore, they were grouped in cluster 6b, showing that light is the predominant environmental signal regulating *LHCs*. Similarly, in *Arabidopsis*, the peak in the expression of many *LHCs* occurs in the early or middle part of the light period [53]. The light pulse that follows the night/darkness induces their transcription, which is modulated by a photoreceptor-triggered transduction pathway [54]. Oscillations in *LHCB* mRNA levels were evident in the monocot rye, with a peak during the light period and rapid decline in the dark [55]. Experiments on barely showed that both LHCA and LHCB apoproteins lagged 4–8 h in their accumulation with respect to the beginning of light exposure [56]. The regulation of these family genes may also be controlled in olive by HY5, which works downstream of PHYB and CRY1. Recent studies showed that the expression levels of *Solanum lycopersicum LHCA* and *LHCB* were negatively regulated when HY5 was silenced but significantly increased in *HY5*-overexpressing lines. The promoters of *LHCA* and *LHCB being* enriched in ACE motifs suggested a direct binding of HY5 to regulate their expression [57]. Similarly, HY5 is required for *LHCA4* upregulation in *Arabidopsis* during the central hours of the daytime, as indicated by the binding to the G-box element in the promoter [58].

The model proposed by Guo et al. (2021) [59] further supports the connection between PHYB and HY5-mediated photosystem functioning, which evidenced that the induction of HY5 by PHYB promotes enhanced iron uptake in tomato, which in turn regulates the electron transport at the PSA and PSB levels. Our RNA-seq data suggest that the genes of olive photosystems, which are upregulated at early morning, are under the indirect control of light-induced photoreceptors.

Many secondary metabolites accumulate in plants in response to several types of stimuli [60], and genetic experiments demonstrated that light represents one of the factors that affects their biosynthesis [61]. Notably, anthocyanin accumulation was increased in tomato plants grown under continuous blue and white light in comparison to those grown under red and far-red light, and high levels of these pigments were observed in *CRY1*-overexpressing lines together with the upregulation of structural genes of anthocyanin biosynthesis. As mentioned above, HY5 acts downstream in the signaling pathway of blue light, and Liu et al. (2018) [62] demonstrated through a ChIP-qPCR assay that its binding to the G-box and ACE motifs in most of the promoters of the anthocyanin biosynthesis genes is required for their upregulation and accumulation. Additionally, the expression level of *HY5* was increased in *CRY1*-OX and decreased in the *cry1* mutant, further supporting the cooperation between them in response to blue light. Intriguingly, in this study, we found that the highest *CRY1* expression coincided with the upregulation of the anthocyanin biosynthesis genes, indicating that this process is HY5-mediated and regulated by CRY1, confirming with what has already been observed in olive fruits, where an additional alternative route that is PHYA-, FHY3-, FAR1-, and PIF3-mediated has been proposed [31].

In fact, HY5 is implicated in the regulation of anthocyanin biosynthesis under far-red light, but the presence of PIF3 is required as a positive component. Both transcription factors bind the promoters of all anthocyanin biosynthesis genes. ChIP analysis showed that there were more ACEs in the promoters enriched by HY5, whereas in the promoters enriched by PIF3, there were more G-boxes [63]. The peak expression of one transcript encoding for PIF3 at ZT0 could explain the combined action of HY5 in promoting the transcription of anthocyanin biosynthesis genes in olive.

In addition to the mentioned mechanisms of the regulation of anthocyanin biosynthesis genes, in rice, the transcription factor HY5 physically interacts with OsBBX14. Transgenic *Arabidopsis* plants overexpressing *OsBBX14* showed upregulation of *AtHY5* as well as the upregulation of *OsHY5* in rice protoplasts, suggesting that the latter is positively regulated by OsBBX14 and both could induce anthocyanin biosynthesis genes in an independent or cooperative manner [64]. The gene expression of the homolog of *AtBBX14* assigned to cluster 4 suggests that the mechanism of regulation of anthocyanin biosynthesis genes found in rice may also occur in olive.

### 3.2. Light and Photoperception Reaction Cascade and FA Accumulation

Interestingly, among the “diurnal genes” showing significant expression during the middle of the day until the dark, we observed a light-mediated gene network potentially implicated in the biosynthesis of oleic acid. Its known that the net fatty acid accumulation in green leaves is largely or entirely confined to the light period and that a redox cascade likely links light and fatty acid synthesis, resulting in the coordination of fatty acid synthesis with photosynthesis [65,66]. Our findings, as well as confirming a similar trend in olive, enabled us to highlight a complex co-regulated gene network involving *FAD2*, a key desaturase for the linoleic acid content in the olive fruit mesocarp [67]; and *FLD*, *COL10*, *SIGC*, and *PLP*, whose abundances are mediated by cryptochromes and phytochromes. In particular, the concurrent decreases in *FLD-*, *COL10-*, *SIGC-*, and *PLP*-domain-containing expressions observed in the comparison between the olive genotypes characterized by difference percentages of oleic and linoleic acid, agree with the *FAD2* downregulation seen in the same samples [31]. For this reason, these results support the hypothesis of a key role of light and the photoperception reaction cascade in the control of FA accumulation and ratios, showing the specific time of the day in which this happens.

### 3.3. Day Length, Circadian Clock, and the Expression of Genes Involved in FA and Phenylpropanoid Pathways

Surprisingly, olive plants survived after three months of LL; in addition, they showed an increase in new sprouts after 30 days under LL (FM) conditions, evidencing intraday expression patten changes for many genes. A total of 125 out of 147 oscillating genes under LD conditions conserved their rhythmicity under LL-FM conditions, suggesting their direct regulation by circadian clock machinery. Among them, tight co-regulation is plausible since a group of genes exhibited a strong peak of expression at ZT12, whereas another group showed marked downregulation at the same time. In addition, among the early morning genes involved in the photoperception and photosynthesis processes, many genes encoding for key structural enzymes in the FA and anthocyanin pathways were found.

After one month of LL exposure (FM), a subset of genes that maintained their LD expression trend were found; among them, many are involved in phenylpropanoid and FA biosynthesis.

Thus, in response to light excess, olive trees likely synchronize the gene expression of photoreceptor-mediated light-sensitive factors as well as anthocyanidin and fatty acid biosynthesis genes, which are together responsible for increased plant growth performance.

After two months under LL (SM) conditions, 94 rhythmic genes showed a similar co-regulation as under LL-FM conditions, although with a lower average expression, showing vegetative plant growth arrest, with a consequent lower chlorophyll content and evident stress symptoms.

## 4. Materials and Methods

### 4.1. Plant Material

One-year-old olive plants of the cultivar ‘Leccino’ were grown in pots under a photoperiod of 16 h light/8 h dark (LD) at a temperature of 23 °C. They were acclimated in growth chambers for 30 days. A light intensity of about 100 µmol m^−2^ s^−1^ was provided by Radium Lampenwerk GmbH (Wipperfürth, Germany) 58 W/865 cool daylight lamps. After the acclimation period, the growth chamber was divided in two compartments separated by a screen, and fifteen out of thirty plants were placed in the compartment exposed to continuous light (LL).

### 4.2. Phenotypic Evaluations

After the acclimation period, all plants were marked with colored wire to follow the vegetative growth (start point, SP). One, two, and three months later (first intermediate point, FM; second intermediate point, SM; and final point, FP) in plants under both LD and LL conditions, the internode length and number were recorded.

Simultaneously, at the same points (FM, SM, and FP), the chlorophyll contents in adult, intermediate, and young leaves sampled from three different branches of each plant were measured using an SPAD-502 Plus instrument (Konica Minolta, Tokyo, Japan). Paired *t*-tests were used to determine statistically significant differences between the means.

### 4.3. Targeted RNA Sequencing

Leaves from three different plants under each grown condition (LD, used as control; LL-FM; and LL-SM) were sampled every 6 h for 24 h using Zeitgeber time (ZT0, ZT6, ZT12, and ZT18), where ZT0 represents the presumptive dawn. Leaves belonging to specific experimental points were pooled, frozen in liquid nitrogen, and stored at −80 °C.

Three independent RNA extractions for each experimental point were carried out with an RNeasy Plant Mini Kit (Qiagen, Hilden, Germany) according to the manufacturer’s protocol. DNA contamination was removed by treatment with an Invitrogen™ TURBO DNA-free™ Kit (Thermo Fisher Scientific, Waltham, MA, USA), and the purity was analyzed via a Thermo Scientific™ NanoDrop™ 2000c Spectrophotometer (Thermo Fisher Scientific, Waltham, MA, USA). The samples with 260/280 and 260/230 nm absorbance ratios greater than 1.8 were used for the following experiments.

RNA integrity was measured via a 2100 Bioanalyzer Instrument with a RNA 6000 Nano Kit (Agilent Technologies, Santa Clara, CA, USA), and the samples with an R.I.N. greater than 8 were used, while RNA quantification was performed using an Invitrogen™ Qubit™ RNA HS Assay Kit (Thermo Fisher Scientific, Waltham, MA, USA) with a Invitrogen™ Qubit™ 4 Fluorometer (Thermo Fisher Scientific, Waltham, MA, USA).

The intraday expression of specific genes was investigated through targeted RNA sequencing (Illumina^®^, San Diego, CA, USA) using a panel employed in our previous study [31]. RNA libraries were prepared via AmpliSeqTM for Illumina (Illumina^®^, San Diego, CA, USA) according to the manufacturer’s protocol. Libraries were assessed via a 2100 Bioanalyzer Instrument using a DNA 1000 Kit (Agilent Technologies, Santa Clara, CA, USA), and DNA quantification was performed using an Invitrogen™ Qubit™ DNA HS Assay Kit (Thermo Fisher Scientific, Waltham, MA, USA) with the Invitrogen™ Qubit™ 4 Fluorometer (Thermo Fisher Scientific, Waltham, MA, USA). Paired-end (PE) complementary DNA (cDNA) libraries were sequenced using a MiniSEQ Instrument (Illumina^®^, San Diego, CA, USA).

The raw reads were archived in the NCBI SRA database under accession number PRJNA1171647.

Quality control checks of the raw sequence data from the Illumina sequencing were performed using FastQC v0.11.9 (Free Software Foundation, Inc., Boston, MA, USA). The adaptors were removed, and the low-quality regions were trimmed (Phred cut-off 20) by using Trimmomatic v0.39 (Free Software Foundation, Inc., Boston, MA, USA). The ‘Leccino’ v. 4 reference genome was used for mapping the reads to predict the genes, although many genes were manually reannotated. The featureCounts function in Rsubread Bioconductor package v2.12.3 was used to evaluate gene expression. The reads were then filtered via expression, excluding any transcript with an abundance less than 10, normalized with the method used in DESeq2 (Median of ratios method) [68,69]. Cosinor analysis was performed with the DiscoRhythm R tool, version 1.22.0 [70].

To evaluate the statistically significant differences in gene expression levels among the four time points within 24 h, one-way ANOVA (Tukey’s pairwise) using R software and Cosinor analysis [71] were performed, and only transcripts with a *p*-adjusted value < 0.05 in at least one of the two methods were considered significant.

The heatmaps were obtained by using the mean value of gene expression according to the average-linkage clustering analysis through the Pearson’s correlations in the heatmapper web tool available at http://www.heatmapper.ca, accessed on 1 January 2025.

## 5. Conclusions

Our study evidenced, for the first time in olive trees, the effects of prolonged light irradiation on the regulation of the key genes involved in pigment and fatty acid accumulation during the day. A correlation between day length and the key role of transcriptional regulation in the expression of many genes involved in photoperception as well as the FA and phenylpropanoid pathways in olive trees was also highlighted and discussed.

Plant growth measurements and transcriptomics analyses of olive plants grown under both LD (light/darkness 16/8 h) and LL (light/light) conditions provided novel insights into understanding the key genes related to FA and phenylpropanoid accumulation under different day lengths.

These relationships could help the scientific community and the researchers involved in breeding programs in the improvement in this species to increase oil quality in the future.

More interestingly, the application of continuous light during plant growth to shorten the juvenile stage was already reported, which is considered a valuable alternative to molecular approaches [4,72,73]. The constitutive or transgenic expressions of key genes related to LL application decreased the time to reach the reproductive stage in poplar, plum, apple, and pear [74,75,76,77]. In olive trees, from seedling stage, the juvenile period can last up to 10 years.

Thus, the present research opens new perspectives for the application of continuous light to reduce the time to flowering in olive trees, demonstrating the capability of this species to survive under LL conditions. Up to three months under LL conditions combined with LD conditions may lead to a faster achievement of the reproductive stage for newly planted orchards, which has important economic implications for farmers.

## Data Availability

The data supporting the conclusions of this article (raw RNA-Seq reads) are available from the National Center for Biotechnology Information (NCBI) Sequence Read Archive (SRA): PRJNA1171647. Supplementary Data associated with this article were uploaded to the submission system.

## Supplementary Materials

The following supporting information can be downloaded at: https://www.mdpi.com/article/10.3390/ijms26010361/s1.
