# Supplementary material for: Circadian- and Light-Driven Rhythmicity of Interconnected Gene Networks in Olive Tree
Source: Int J Mol Sci. 2025 Jan 3;26(1):361. doi: 10.3390/ijms26010361 (PMC11719796; doi:10.3390/ijms26010361)

**Supplementary information**

**Different circadian and light driven rhythmicity of interconnected gene networks in olive tree**

Ivano Forgione^1^, Tiziana Maria Sirangelo^1^, Gianluca Godino^1^, Elisa Vendramin^2^, Amelia Salimonti^1^, Francesco Sunseri^3^ and Fabrizio Carbone^1*^.

*^1^Research centre for Olive, Fruit and Citrus Crops, Council for Agricultural Research and Economics (CREA) - Via Settimio Severo, 83, 87036 Rende (CS), Italy*

*^2^Research centre for Olive, Fruit and Citrus Crops, Council for Agricultural Research and Economics (CREA) - Via di Fioranello 52, 00134 Roma, Italy*

*^3^Department Agraria, University Mediterranea of Reggio Calabria, Località Feo di Vito, 89124 Reggio Calabria (Italy)*

*Correspondence: fabrizio.carbone@crea.gov.it

**Tables (Supplementary Tables.xlsx)**

**Table S1.** Vegetative growth in terms of length of new sprouts (cm) and number of internodes (n.).

**Table S2**. Chlorophyll content (SPAD) in adult, intermediate and young leaves.

**Table S3** Function, annotation and normalized raw sequencing data of all replicates are reported for each locus.

**Table S4** Summary of reads filtering and alignment processes.

**Table S5** Summary of the 147 significant loci in LD clustered according to the up-regulation within the 24h. Mean expression of three replicates for ZT0, ZT6, ZT12, ZT18 and p-value of Cosinor and ANOVA analysis are reported.

**Table S6** Summary of the 125 significant loci in LL-FM. Mean expression of three replicates for ZT0, ZT6, ZT12, ZT18 and p-value of Cosinor and ANOVA analysis are reported.

**Table S7.** Summary of the 94 significant loci in LL-FM. Mean expression of three replicates for ZT0, ZT6, ZT12, ZT18 and p-value of Cosinor and ANOVA analysis are reported.

**FIGURES**

**Fig. S1:** Representative pictures of olive plants grown under LD (Control Plants), LL-FM, LL-SM and LL-FP conditions. Scale 1:5.


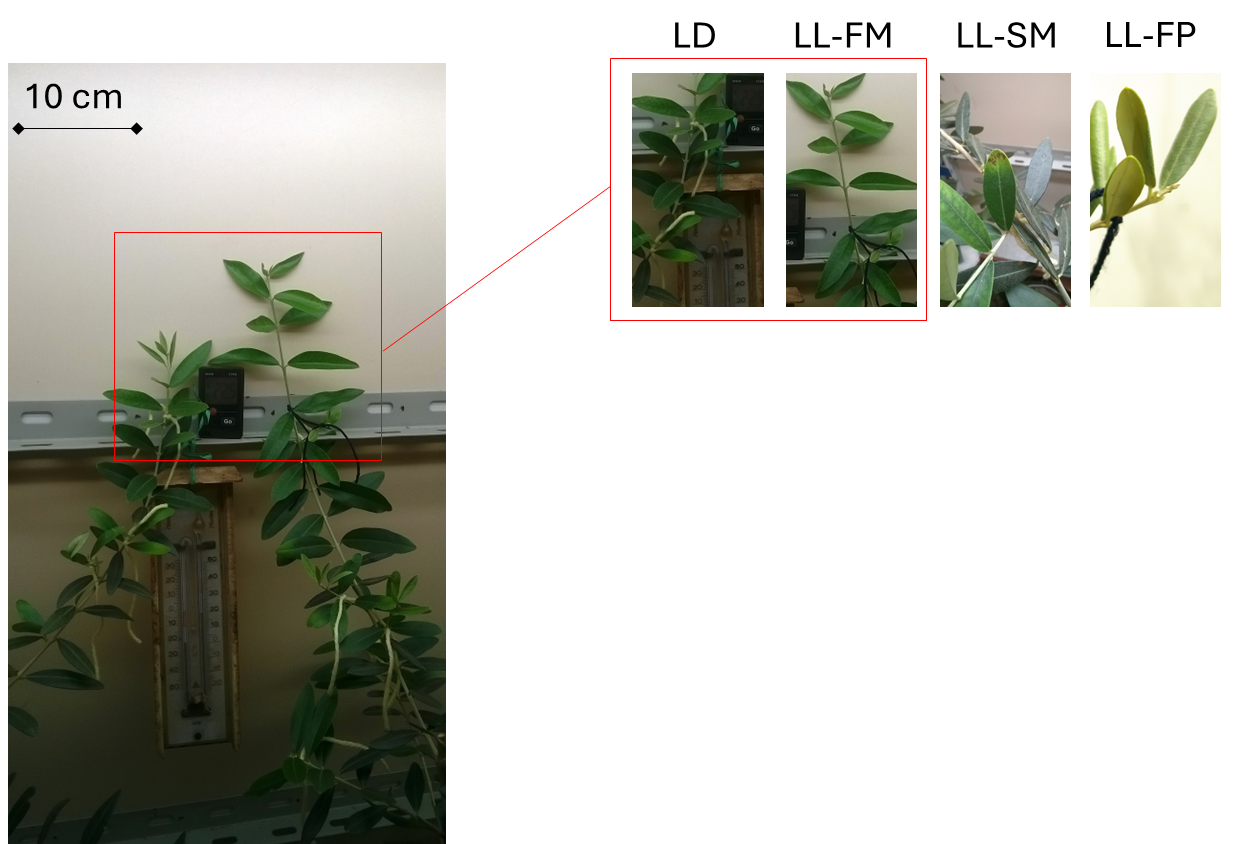

Supplement: Supplementary file 1 [file ijms-26-00361-s001.zip › Supplementary_data.docx]
